# Effects of Point-Of-Care Testing in General Practice for Type 2 Diabetes Patients on Ambulatory Visits and Hospitalizations

**DOI:** 10.3390/ijerph17176185

**Published:** 2020-08-26

**Authors:** Troels Kristensen, Kim Rose-Olsen, Christian Volmar Skovsgaard

**Affiliations:** 1 DaCHE, Department of Public Health, Faculty of Health Sciences, University of Southern Denmark, J.B. Winsløw vej 9, 5000 Odense C, Denmark; krolsen@sdu.dk (K.R.-O.); chsko@sam.sdu.dk (C.V.S.); 2 Research Unit of General Practice, Faculty of Health Science, University of Southern Denmark, J.B. Winsløw vej 9, 5000 Odense C, Denmark

**Keywords:** point-of-care testing (POCT), diabetes, general practice, HbA1c, patient data, hospital admissions, ambulatory visits, difference-in-differences (DID) models, event study analysis

## Abstract

Point-of-care testing (POCT) of HbA1c means instant test results and more coherent counseling that is expected to improve diabetes management and affect ambulatory visits and hospitalizations. From late 2008, POCT has been implemented and adopted by a segment of the general practices in the capital region of Denmark. The aim of this study is to assess whether the introduction of POCT of HbA1c in general practice (GP) has affected patient outcomes for T2 diabetes patients in terms of hospital activity. We apply difference-in-differences models at the GP clinic level to assess the casual effects of POCT on the following hospital outcomes: (1) admissions for diabetes, (2) admissions for ambulatory care sensitive diabetes conditions (ACSCs), (3) ambulatory visits for diabetes. The use of POCT is remunerated by a fee, and registration of this fee is used to measure the GP’s use of POCT. The control group includes clinics from the same region that did not use POCT. The sensitivity of our results is assessed by an event study approach and a range of robustness tests. The panel data set includes 553 GP clinics and approximately 30,000 diabetes patients from the capital region of Denmark, observed in the years 2004–2012. We find that voluntary adoption of POCT of HbA1c in GP has no effect on hospital admissions and diabetes-related hospital ambulatory visits. Event study analysis and different treatment definitions confirm the robustness of these results. If implementation of POCT of HbA1c improves other parts of diabetes management as indicated in the literature, it seems worthwhile to implement POCT of HbA1c in the capital region of Denmark. However, doubts around the quality of POCT of HbA1c testing and a desire to capture data at central labs may prevent implementation of more value based HbA1c testing.

## 1. Introduction

Timely measurement of HbA1c, which reflects blood sugar levels over the previous three months, is central to the control of T2 diabetes. These measurements are important for both the quality of diabetes care, patient outcomes, consumption of health care services and pharmaceuticals and the subsequent cost of care [1,2,3]. In general practice (GP) clinics, HbA1c control is usually performed on venous blood that is sent to a central hospital laboratory where the response is typically available after 1–2 days. Guidelines recommend that HbA1c should be measured 2–4 times per year [4]. However, the guidelines are not met for all T2 diabetes patients. For example, due to undersupply by GPs or poor self-management by the patients [5,6]. Poor self-management may be related to such issues as reduced ability to receive care due to comorbidities, psychiatric disease and social problems [7].

One approach to increase compliance and improve outcomes may be to introduce point-of-care tests (POCT) of HbA1c in GP clinics [8]. HbA1c by POCT provides rapid (5–7 min) finger-stick capillary blood collection, to facilitate medical decision making [9]. POCT also saves time (and costs) both for healthcare professionals and patients, catalyze greater engagement with patients, better adherence and compliance, reduce demand for self-disciplining, increased patient loyalty and patient satisfaction (e.g., more pleasant) [10,11,12,13]. POCT of HbA1c is recommended by the American Diabetes Association (ADA) for monitoring patients with diabetes.

An expected central outcome is improved HbA1C levels [14] and changes in hospital activity [15,16]. Hence, we focus on patient outcomes in terms of admissions and ambulatory hospital care and follow [17,18] who use hospitalizations and diabetes-related ambulatory visits to measure diabetes outcomes. On one hand, it can be argued that improved monitoring via POCT may increase ambulatory and hospital admissions. In particular, among patients that otherwise are difficult to monitor, e.g., for vulnerable and complex patients that do not visit their GP on a regular basis. On the other hand, POCT may also lead to reduced ambulatory and hospital activity due to better glycemic control in GP. Patzer, for example, found that implementation of POCT reduced the number of required visits by 80% and that 82% of patients (vs 13% prior to POCT) were able to discuss their HbA1c values with the treating physician immediately at first visit [19]. Thus, it is not well understood whether patients with diabetes monitored in GP clinics via POCT experience more or less diabetes related hospital activity. The aim of this study is to assess the effect of introduction of POCT of HbA1c on hospital admissions and ambulatory visits in Denmark from 2009–2012.

## 2. Methods and Data

Ultimo 2008, Danish regulators agreed to create a new national framework for the remuneration of POCT of HbA1c for monitoring diabetes patients in general practice. It was agreed that general practitioners should be paid a new fee (of Danish Krone 115.97 or 15.49 €) per POCT of HbA1c. An expected effect was reduced use of hospital and private walk-in laboratory capacity, GP visits and reduced need for transportation of blood samples to central laboratories. Unfortunately, the tariff was only implemented in the capital region, where just a part of the GP clinics started using this new option.

This voluntary uptake of POCT by some GPs and lack of implementation by other GPs in the capital region represents a natural experiment. This type of experiment is often recommended as a way of analyzing and understanding the impact of policy changes [20]—e.g., on outcomes such as hospital activity. One characteristic of a natural experiment is that exposure to the technology cannot be manipulated by investigators [21,22]. Thus, when special care is taken (e.g., in designing, reporting and interpretation of the exposed and unexposed population), it allows us to compare effects if observational data on exposure, outcomes and potential confounders are obtained.

This study applied a difference-in-differences (DID) method to analyze GP clinics which implemented POCT versus a control group of non-implementers and estimated the effects of POCT on the average rates of hospitalizations and ambulatory hospital visits [20,23,24]. The special fee-for-service fee that was introduced for POCT of HbA1c ultimo 2008 was applied as proxy measure for POCT of HbA1c among diabetes patients in general practice [25]. POCT clinics were defined as clinics using POCT at least once during a year.

### 2.1. Identification and Estimation Strategy

To identify the average treatment effect (ATT) of POCT on hospitalizations and ambulatory visits and to account for selection bias, we used a DID framework with a continuous treatment variable and included GP and year fixed effects as well as time-varying control variables. Hence, we estimated the effect of POCT on outcomes for GP clinics that used POCT in a given year compared to clinics that did not. To take GP variation into account unrelated to the introduction of POCT, we included GP fixed effects (for further technical details see Appendix A).

### 2.2. Dependent Hospital Outcome Variables and Covariates

The treatment effect of POCT on diabetes patients was assessed for three GP clinic level outcome variables: (1) average diabetes admission rates, (2) average ACSC diabetes admission rates and (3) average outpatient visit rates for diabetes. ACSC admissions were added because ACSC admissions are widely used as indicators of primary care outcome [26].

The study controls for potential selection bias via time-varying covariates to obtain a conditional common pretreatment trend in hospitalizations and ambulatory visits as the implementation of POCT was voluntary. These covariates are used to adjust for differences between the control and the treatment group of GP clinics around the time POCT was implemented. The vector of control variables includes three subsets of clinic covariates: (a) the diabetes management control variables for GP clinics (the clinics’ proportion of diabetes patients, the share of patients registered on a special diabetes bundle payment fee, the average number of GP visits and the proportion of the diabetes patients with diabetes experience (diabetes age >5 years)); (b) the average GP clinic level morbidity burden measured via the aggregated Charlson index for all patients linked to the clinic. Diabetes was excluded from the Charlson index to avoid endogeneity regarding the outcomes of interest; and (c) the socioeconomic mix of patients listed in each GP clinic (including the proportion of patients over 65 years, the proportion of unemployed patients, the proportion of singles and the average family income). The clinic-level diabetes management control variables (a) were included as proxies for GPs with special interest in and knowledge about diabetes. The average Charlson index (excluding diabetes) and the proportions of socioeconomic characteristics were used to adjust for dynamic differences in patient morbidity burden and socioeconomic background. To explore the contribution from each set of covariates and combinations of these three subsets we estimated three models: Model 1 includes the key term of interest (the treatment interaction term), Model 2 adds control variables from (a), and Model 3 adds control variables from (b) and (c).

### 2.3. Treatment Group, Control Group and Introduction Years

The flexible “treatment” group used in the DID analysis was defined as clinics that introduced POCT during the period 2009–2012 and continued using it all subsequent years after their introduction through to 2012. Hence, the treatment definition allowed for flexible introduction to include more GP clinics in the analysis. Table 1 illustrates how we used an indicator variable $I_{it}$ to capture all combinations of possible introduction years ($T_{i}$) and years of observation ($t_{i}$).

For instance, clinics that introduced POCT in 2010 are included in the treatment group in the Years 2010–2012 while clinics that introduced POCT in 2011 are included in the treatment group in the Years 2011–2012. This flexible approach made it possible to include GP clinics at the time when they introduced the technology. The GPs that used a central hospital laboratory test or walk-in labs and never used POCT were applied as the control group. Hence, the POCT clinics and the control group were exposed to the same “competing” explanations for change in outcomes.

Next, the POCT clinic treatment measure ($C_{it}$) was defined as the share of diabetes patients linked to the GP clinic which were treated with POCT in a given year. This share rather than a dummy approach allows us to account for the intensity in the use of POCT, which is expected to be positively related to the treatment effect [27].

### 2.4. Yearly Point Estimates and Sensitivity Analysis

To visualize our findings and test the sensitivity of the effects of POCT on hospital activity, we used an event study approach that investigates and visualizes the effect for each year before (testing for falsification) and after the flexible introduction year (testing for treatment effect) [23]. We defined the variable $k_{i}$ for each GP i, where $k_{i}$ represents the number of years before and after the introduction year of POCT at GP i. Table 2 shows these years before and after introduction of POCT (ki) for each introduction year (Ti) and year (ti).

For years before the introduction in Table 2, $k_{i}$ is negative. When $k_{i}<-1$ in (i.e., the south–west of Table 2), the confidence interval around the point estimates for each, $k_{i}$ of the estimated DID model was used to test the parallel assumption. This means tested in the sense that after taking the covariates into account, we should see no difference in outcomes (relative to the baseline year) between the treatment and the control group prior to the introduction. The flexible reference year is always the year before $GP_{i}$ introduces POCT, $k_{i}=-1$. This year (rather than $k_{i}=0$) was used to ensure that the GP did not use the equipment in the reference year. Hence, all estimates in the event study should be interpreted relative to that flexible reference year. As $GP_{i}$ introduces POCT at some point throughout the year $k_{i}=0$, the estimate for $k_{i}=0$ should be interpreted in that context as effects may not have occurred fully yet (for further technical details see Appendix A).

### 2.5. Robustness Tests

To investigate the robustness of our results, we used (a) an alternative standard dichotomous treatment measure instead of the continuous measure; (b) a treatment definition that only included the first mover clinics that introduced POCT in 2009 and (c) performed subgroup analysis for different sets of patient types.

The alternative dichotomous treatment measure defined GP clinics as treated if they used at least 5 POCTs during a year in all subsequent years after the clinic’s introduction. The model of the early adopters included the clinics that introduced POCT in 2009 and used it throughout the remaining period of observation. This test was performed to explore the effects of POCT among patients of the early adopters, as these clinics used POCT more intensively and for the longest period potentially allowing us to estimate more long run effects. These GP clinics were also likely to be those that have a special interest in diabetes and hence may constitute a potential selection problem in our main model. Finally, subgroup analyses for selected types of patients (high/low Charlson Index, high/low educational level, above/below age 65 and Danish/foreign ethnicity) were undertaken to explore the robustness of the results to these subgroups. The selected subgroups were based on [25].

## 3. Data, Implementation of POCT and Descriptive Clinic Characteristics

### 3.1. Data for T2 Diabetes Patients and Clinics

This study used gross clinic data as unit of analysis. This means a panel data set of patient data clustered at GP clinic level covering the years 2004–2012 for a cohort of T2 diabetes patients from the capital region of Denmark. The cohort of T2 diabetes patients was defined by the algorithm of the Danish Diabetes Register based on the Danish Drug Register, the Danish Health Service Register and the National Patient Register. Patients were required to be above 18 years of age, alive in 2012 and living in Denmark. Furthermore, at least one out of the following three criteria had to be met in a given year: (1) The patients had redeemed at least one prescription for antidiabetic drugs with ATC code A10A* or A10B* (*: including subgroups). ATC code A10BA02* (metformin) was excluded for women between the ages of 20–40 (gestational diabetes); (2) The patients had received at least three blood sugar or HBA1c tests in the primary care sector 3) The patients were registered with one of the following ICD10 codes in the hospital sector: E11*, E12*, E13*, E14*, O24* or H360*. Patients who in any given year received medicine related only to T1 diabetes were excluded (see Appendix B and Appendix C for further details).

To be included in the cohort, patients had to be identified by the algorithm described above at least once during the period 2004–2006. The cohort was identified in the period leading up to the analysis period 2006–2012 to allow us to understand and explore the difference in the control and treatment group before and after the intervention, without the pre-introduction period being influenced by accession and attrition. To avoid including patients without diabetes, individuals who were identified only once during the entire period 2004–2012 were excluded. In total, the cohort consisted of more than 30,000 T2 diabetes patients. Each patient was linked to the GP clinic they used most frequently.

Data approval for conducting the study was provided by the Data Protection Agency (ref. nr 17/6021). An anonymized id number using public health services was used to merge data at the individual level from the Danish administrative registers.

### 3.2. Uptake Among GP Clinics, 2008–2012 and the Share of T2 Diabetes Patients Receiving POCT

Table 3 shows the share of clinics in the capital region that started using POCT at least once since the introduction ultimo 2008.

The implementation rate increased to approximately 14% in the first year after the introduction. Subsequently, the uptake increased in a regressive way until 2012, where the share of GPs using POCT was approximately 39%. Table 4 shows the POCT clinic share of diabetes patients who received POCT.

The share of diabetes patients who received at least one POCT during the introduction year (k=0) was approximately 24%. In the first to third year (k≥1), the GPs in POCT clinics reached a level around 50% for the share of diabetes patients who should receive POCT.

### 3.3. Descriptive Clinical Characteristics

Table 5 shows the mean values for the outcome variables and time-varying covariates for the POCT group (231 clinics) and the control group (322 clinics) at the clinic level before the introduction of POCT in 2008. These values show how the two groups differed in terms of descriptive variables.

The control group had a higher proportion of diabetes related outpatient visits, a lower proportion of diabetes patients, a higher share of patients with diabetes age above 5 and a higher share of single patients. To take these differences into account in our analysis we control for the differences in our regression models.

## 4. Results

### 4.1. The Effect of POCT of HbA1c

Table 6 shows the effect of POCT of HbA1c on diabetes-related admissions (columns 1–3) on diabetes-related ACSC admissions (columns 4–6) and ambulatory care activity (columns 7–9). The results do not support the hypothesis that POCT should reduce hospitalizations and/or ambulatory care visits. For all models, this evidence is reflected by the fact that none of the estimated treatment effects are significant.

Most the time-varying covariates show limited impact on the outcomes in the DID model. Only covariates that vary between the treatment and control group around the time of the introduction are likely to be significant. The only covariate that show a significant association with the outcomes is the Charlson index. However, if we compare the full model with the restricted model, the inclusion of the Charlson index does not alter the significance or the magnitude of the effect of POCT.

### 4.2. Event Study Analysis: Dynamic Yearly Estimates of the Effects of POCT of HbA1c

**Figure 1:**
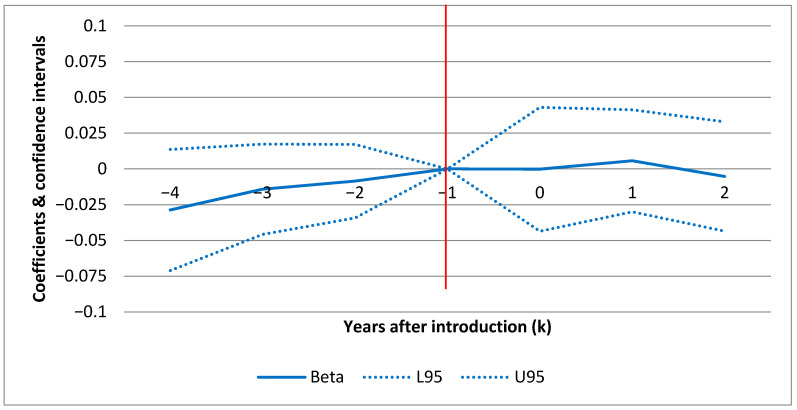
Yearly effects of POCT of HbA1c on admissions, point estimates. Note: dotted lines represent 95% confidence intervals. The model includes year and clinic fixed effects and the full set of covariates from Table 6.

**Figure 2:**
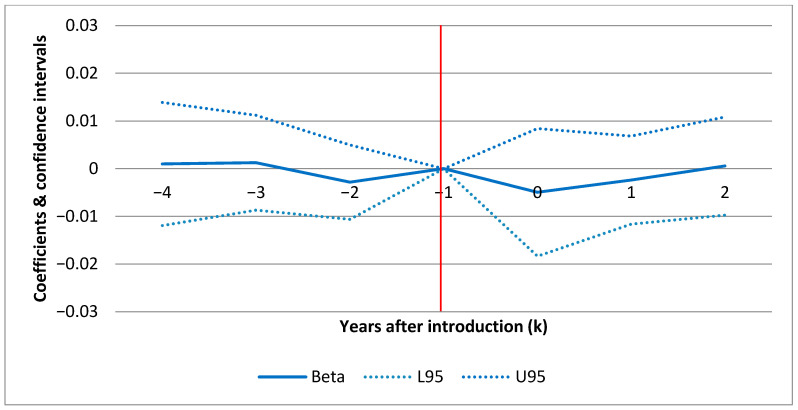
Yearly effects of POCT of HbA1c on ambulatory care sensitive diabetes condition (ACSCs) admissions, point estimates. Note: The dotted lines represent 95% confidence intervals. The model includes year and clinic fixed effects and the full set of covariates from Table 6.

**Figure 3:**
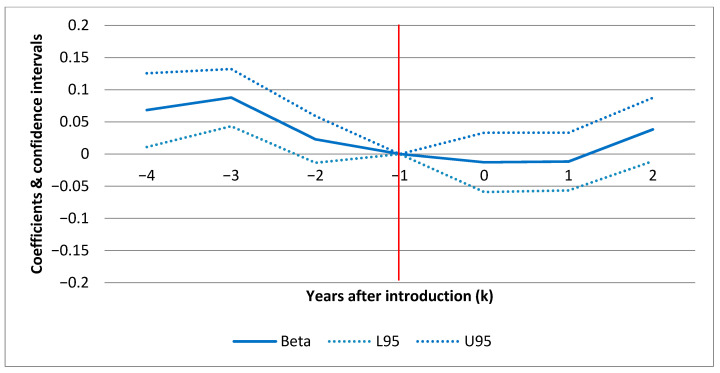
Yearly effects of POCT of HbA1c on ambulatory care, point estimates. Note: dotted lines represent 95% confidence intervals. Model includes year and clinic fixed effects and the full set of covariates from Table 6.

Figure 1 (F1), Figure 2 (F2) and Figure 3 (F3) show the confidence intervals (dotted lines for upper (U95) and lower (L95) 95% CI) for yearly point estimates of the effect of POCT of HbA1c on standard admissions, ACSC admissions and ambulatory care visits(respectively) for each year before and after the introduction. The results in F1–F3 are based on the full models in Table 6 (columns 3, 6 and 9) including all set of covariates a–c) but estimating yearly effects rather than the average effect.

In each figure (F1–F3) the horizontal axis displays the number of years before and after introduction as illustrated by the year markers $k_{i}$ in Table 2. The results are all relative to the effect in the baseline year ($k_{i}=-1$) before the individual clinics introduced POCT. This means that the CIs show the effect for each year relative to the pre-introduction year (red line intersecting [−1,0]). For $k_{i}>0$, a positive estimate indicates a treatment effect in the given year. F1–F3 show the effect over time for each hospital outcome. For example, this allows us to investigate whether an early effect declines over time or vice versa. In F1 and F2, there are no sign of any patterns over time. For ambulatory care (F3), there is a slight increase in the second year after introduction. However, as shown in Table 6, where the model estimates differences between the average post- and pre-introduction periods rather than yearly estimates, the average effect on ambulatory care is negative and insignificant.

### 4.3. Parallel Assumption

For both types of hospitalizations, the parallel assumption in F1 and F2 seems to be fulfilled. The justification is that there is no difference in outcomes (relative to the baseline year) between the treatment and the control group prior to the POCT introduction after taking into account covariates (zero within CI prior to intro). For ambulatory care visits, there are minor exceptions for single years ($k_{i}=-4\;\mathrm{and}\;k_{i}=-3$). This means that 4 and 3 years prior to the introduction, patients from the POCT clinics had more ambulatory care visits in contrast to the control group.

### 4.4. Robustness

We tested a binary treatment definition. The estimates were similar to the results for the continuous definition. This indicates that the results are not driven by the choice of treatment measure. Next, we tested a model that only included GP clinics in the treatment group that introduced POCT in the first possible year (2009). This model revealed a borderline significant reduction in ambulatory care due to the use of POCT when not all covariates were included. However, the parallel assumption was not fulfilled for this specific group of clinics. The subgroup sensitivity analyses did also not show treatment effects of POCT of HbA1c on hospital activity for any of the eight subgroups (results available upon request).

As an adjustment to the treatment measure, a robustness test also explored the number of POCT per T2D patient on the patient list. This alternative measure adjusted for variation in the frequency with which the GPs used POCT. It was highly correlated (correlation coefficient ρ=0.96, p<0.001) with the (unadjusted) treatment measure. Therefore, this adjustment did not change the results (results available upon request).

Finally, it was a part of the empirical strategy to control for GP list size. The average list size in the treatment group was larger than the list size in the control group. However, as the GP list size is very stable over time it did not affect the estimated effects of POCT (results available upon request).

## 5. Discussion

This study shows that three years after the introduction of FFS for POCT in the capital region of Denmark 39% of GPs used POCT to measure HbA1c and that the share of patients having HbA1c measured by POCT was 50%. The results of this DID analysis of the natural experiment show that POCT of HbA1c does not affect the average number of ambulatory visits and hospital admissions among T2 diabetes patients in the capital region. This finding is important since the technology has already been argued and shown to reduce operations cost and improve patient satisfaction and has been recommended by recognized stakeholders such as the American Diabetes Association [28,29]. In addition, the policy environment has changed recently. In 2017, even more treatment of selected chronic conditions (COPD and T2 diabetes) in Denmark were shifted to general practice, and in 2018, a new fixed comprehensive incentive structure was implemented for type 2 diabetes patients in Danish general practice. These policy changes have made the use of POCT even more policy relevant. For example, to limit higher workload in general practice associated with these changes.

The finding of no impact on hospital activity is robust to a number of robustness checks and sensitivity analyses. The results only reveal indications of a reduction in outpatient activity for the subgroup of the early adopter clinics that had been using POCT for all four subsequent years. This could indicate that an effect takes longer time to materialize than our follow-up period allows or that the early adopters are different.

This study did not find a decrease in hospital admission and ambulatory activity as it was the case in German [14]. One reason may be that Danish T2 diabetes patient were well covered by GPs in the years analyzed. Another reason may be difference in the available and applied data. Even though, we can only link POCT to the GP clinic (rather than the individual GP’s authorization number), we have detailed data such as the Charlson index about patient composition.

It can be discussed whether to use gross clinic data or individual patient data as the unit of analysis. On one hand, it can be argued that stochastic patient specific data increases the number of observations and the statistical power to undertake significant tests [30]. On the other hand, the hospital outcomes at patient level in this study are typically dummy variables reflecting dichotomous outcomes (e.g., visit/no visit). A central part of these patient observations would be zero-observations leading to a zero-inflated regression model problem. One approach would be to use a regression model based on a zero-inflated probability distribution. Still, this type of model is not well suited to include fixed effects due to the “incidental parameter problem” [31]. Without fixed effects we would not be able to adjust for unobserved clinic characteristics which is considered important to control for GP selection into POCT usage.

### 5.1. Organization of Diabetes Treatment

Stakeholders in the pathway comprise both in- and outpatient care facilities, general practice, independent laboratories and hospital laboratories. Since 2008–2009, each of the five regions; which have political responsibility for operations and capacity planning, has developed pathway programs for type 2 diabetes including stratification of patients according to the degree of severity and the related care need. The standard pathway comprises yearly complication screening, stratification and revision of treatment plans by primary care or outpatient hospital clinics.

From 2009–2012, most T2 diabetes patients were diagnosed and treated in general practice clinics. It was considered relevant to keep T2 diabetes patients treated at the lowest cost level. However, the presence of vulnerable patients, language barriers, cultural differences, social and compliance problems often makes it necessary to create individualized pathways. Therefore, patients with certain diabetes management problems who had progressed to more severe disease stages were treated in the hospital sector or were so-called shared care patients.

In the literature, there is no clear evidence showing that POCT improves diabetes care in terms of hospital activity as studies show both improvement and/or no effect. One review e.g., shows that HbA1c testing at the point of care, improves clinical outcomes [32]. Another review shows that a regular source of primary care and a well-controlled HbA1c level decreased the likelihood of hospitalization. Higher patient age, increased comorbidity and lower socioeconomic status were related to higher hospitalization. Gender and health-related lifestyle showed no relationship [14]. In a third review, Al-Ansary et al. found that there was a nonsignificant reduction in the HbA1c level in the POCT group compared to the control group and absence of evidence in clinical trial data for the effectiveness of POCT for HbA1c in the management of diabetes [33]. Finally, Gialamas et al. did not provide robust evidence that POCT in general practice improves patient health outcomes [34].

### 5.2. Selection Bias

By using a fixed population of patients with diabetes defined in the pre-analysis period 2004–2006 (before the natural experiment), we eliminate the problem of patient selection into the treatment group in the later phases of the experiment. However, GPs that voluntarily choose to use POCT may be subject to self-selection. An obvious reason could be that GPs in POCT clinics are more interested in diabetes and have better performing diabetic patients due to the GPs being more competent with diabetes management. Another reason could be that larger clinics may adopt and use new technology faster. A negative treatment effect may be the result of selection due to voluntary adoption rather than evidence regarding efficacy of POCT. We find indications of selection bias for one of our three outcomes (outpatient visits) three to four years prior to the introduction, but in the second year prior to the introduction there is no sign of selection as the treatment and control groups are similar after taking into account control variables. Hence, overall, the event study does not indicate that selection is a problem after controlling for a number of time-varying observable covariates as well as time-invariant unobservable variables controlled for by the fixed effects. For instance, including GP list size did not alter the results since the GP list size is very stable over time and hence, the fixed effects captured the main part of this variation across clinics.

The absence of the implementation of the POCT of HbA1c in the four other regions has been analyzed and discussed by health care professionals [25]. However, the regulator’s arguments for lack of implementation have not been explicitly communicated. Regional decisions regarding implementation may, for instance, be related to: (a) medical opinions in the individual regional laboratory committees or other regional preferences regarding the use of hospital laboratories capacity, (b) the transportation capacity between clinics and laboratories and (c) the preferences for central testing and analysis, for instance, to ensure the quality of tests and that the test results are registered centrally for R&D purposes. We expect that this study can help the regions to an improved understanding of the effect of POCT of HbA1c technology and motivate more explicit communication regarding these decisions.

### 5.3. Limitations

The availability of HbA1c tests in GP clinics was promoted to the patients via their GPs and their staff during visits such as yearly control visits. It is likely that some patients may have been informed by other patients or stakeholders such as patient organizations and created a demand for POCT. Unfortunately, it is unknown what part of all GPs were aware of the new technology. Those GPs who were aware of the option to use POCT could use it for monitoring of all their patients with diabetes. After the introduction of POCT of HbA1c in GP clinics in late 2008, the uptake of the technology increased steadily, reaching almost 40% of the clinics in 2012. Within the second year of implementation, clinics reached a utilization rate of approximately 50% of diabetic patients after which the rate was steady. This indicates that GPs do not intend to apply POCT for all diabetic patients. This is a limitation of the study because the estimated average treatment effects are based on the use of POCT in this subsample of clinics where POCT is used on approximately only half of their diabetic patients.

Furthermore, the POCT fee is only the best available proxy for the use of POCT of HbA1c in primary care. How accurate the register data reflect reality is unknown without validation, and hence, we rely on the GP’s economic incentive to claim the fee. To identify the patients with diabetes, we used a validated algorithm used in other studies, the Danish Diabetes Register and the WHO. Nevertheless, an algorithm is only a proxy of the real cohort of patients with diabetes.

Diabetes is a chronic and slowly progressive disease. It may be optimistic to expect an effect on hospital admission rates within our time frame of up to four years. However, other studies on diabetes management tend to find changes in hospitalization rates within the same timeframe [14,17]. In this light, we find the present timeframe to be useful for assessing the role of POCT on hospital admissions. Laboratory results of HbA1c changes and diabetes complications registered in primary care and hospitals are other alternative outcomes in diabetes management. For instance, visits to Ophthalmic Medical Practitioners (OMP), visits to foot specialists and diagnoses related to micro- or macro–vascular complications. Unfortunately, data on laboratory results of HbA1c changes and related clinical outcomes such as HDL, LDL, weight and smoking status are not collected from Danish GPs. These data and ICPC-2 coding on diabetes patients (e.g., vascular complications) were captured in a central Danish quality of care database and used for quality of care reports between 2005–2014 until the data base was closed for legal reasons [35,36]. Regrettably, this failure to report data from general practice still leave large gaps in the stakeholder’s overview of diabetes treatment in Denmark.

Yet another alternative outcome is the cost of POCT of HbA1c services. We find that POCT of HbA1c does not change the extend of outpatient and inpatient care in hospitals. However, in the future, it is considered relevant to analyze the effect of POCT of HbA1c on resource use data.

To understand the role of POCT at the patient–health system interface, more research is needed to ascertain the effects on outcomes, prescription behavior and distributional implications across socioeconomic groups of patients and how the policy environment and health care practices influence the usefulness of POCT [13].

## 6. Conclusions

This study shows that voluntary adoption of POCT of HbA1c in GP clinics has no effect on diabetes-related hospital admissions and hospital ambulatory visits. Hence, if the implementation of POCT of HbA1c as stated in the literature improves other parts of diabetes management such as patient adherence, patient satisfaction and clinical operations for GPs, it seems worthwhile to implement POCT of HbA1c. However, doubts around the quality of POCT of HbA1c testing, cost of alternative laboratory tests and a desire to capture data at central labs may prevent implementation of more value based HbA1c testing.

## Figures and Tables

**Table 1:** Flexible point-of-care testing (POCT) introduction year.

		Year ($t_{i}$)						
	$I_{it}$	2006	2007	2008	2009	2010	2011	2012
**Intro year ($T_{i}$)**	**2009**	0	0	0	1	1	1	-
	**2010**	0	0	0	0	1	1	1
	**2011**	-	0	0	0	0	1	1
	**2012**	-	-	0	0	0	0	1

Note: Table shows for each year $t_{i}$ whether a clinic that introduced POCT in year $T_{i}$ is included in the treatment group.

**Table 2:** Year markers used to estimate dynamic effects of POCT, 2006–2012.

		Year ($t_{i}$)						
	$k_{i}$	2006	2007	2008	2009	2010	2011	2012
**Intro year ($T_{i}$)**	**2009**	−3	−2	−1	0	1	2	-
	**2010**	−4	−3	−2	−1	0	1	2
	**2011**	-	−4	−3	−2	−1	0	1
	**2012**	-	-	−4	−3	−2	−1	0

Note: Table shows for each year $t_{i}$ the number of years $k_{i}$ before and after the introduction of POCT in the clinic in year $T_{i}$. When $t_{i}$ is the same as the introduction year, then $k_{i}=0$.

**Table 3:** Uptake of POCT of HbA1c among general practice (GP) clinics in the capital region.

Year	2008	2009	2010	2011	2012
Share of clinics using POCT	0.5%	14.8%	26.0%	32.8%	38.7%

**Table 4:** POCT clinic shares of diabetes patients receiving POCT after flexible introduction.

Years since introduction at GP (k)	−1	0	1	2	3
Share of clinics using POCT	0%	24%	47%	51%	51%

Note: The share of clinics using POCT shows the average share of the POCT clinic’s patients who received POCT of HbA1c in the year before introduction (k=−1), in the introduction year (k=0) and in the three years following the introduction (k=1, k=2, and k=3).

**Table 5:** Descriptive clinic characteristics for the treatment and control groups before the introduction.

	2008		
	Control Group	POCT Clinics	*p*-Value
	(1)	(2)	(3)
**Outcome variables (rates)**			
Diabetes inpatients admissions	0.100	0.100	0.999
Diabetes ACSC inpatient admission	0.011	0.009	0.320
Diabetes outpatient visits	0.381	0.308	**0.000**
**Diabetes characteristics & management**			
Proportion of diabetes patients	0.023	0.028	**0.000**
Share of clinics, bundle payments	0.044	0.062	0.422
Average number of GP consultations	6.770	6.998	0.212
Share of patient’s diabetes age >5	0.544	0.515	**0.011**
**Morbidity burden**			
Charlson index (excl. diabetes)	0.208	0.199	0.415
**Socioeconomic proportions**			
Proportion of elderly patients, age >65	0.502	0.490	0.330
Proportion of unemployed patients	0.042	0.039	0.495
Patient family income, (DKK)	204,129	204,816	0.894
Proportion of single patients	0.394	0.371	**0.044**
N	322	231	

Note: Treatment group includes clinics that became POCT clinics during the period from 2009 to 2012. Group differences significant at the 5% level are in bold. GP: General Practice.

**Table 6:** Average effect of POCT of HbA1c for standard difference-in-differences (DID) model with continuous treatment variable.

Dependent Variable:	Diabetes Admissions			ACSC Diabetes Admissions			Ambulatory Care, Diabetes		
	(1)	(2)	(3)	(4)	(5)	(6)	(7)	(8)	(9)
POCT * I^year^	0.00957	0.00863	0.00999	–0.00198	–0.00220	−0.0023	−0.0152	−0.0158	−0.0131
	(0.70)	(0.62)	(0.75)	(−0.62)	(−0.68)	(−0.71)	(−0.87)	(−0.90)	(−0.75)
**Diabetes characteristics & management**									
Proportion of diabetes patients		1.116	1.075		0.315	0317		1.185	1.439
		(1.30)	(1.23)		(1.64)	(1.64)		(0.88)	(1.11)
Share of bundle payments		0.00062	0.00232		0.00205	0.0022		0.0109	0.0123
		(0.09)	(0.35)		(0.99)	(1.06)		(1.25)	(1.41)
Number of GP consultations		0.00335	0.00256		–0.00014	–0.00019		–0.00208	–0.00226
		(1.52)	(1.23)		(−0.28)	(−0.36)		(−0.67)	(−0.79)
Share of diabetes patients age >5		−0.0366	−0.055		–0.00507	–0.00625		−0.0866	−0.0864
		(−0.45)	(−0.71)		(−0.30)	(−0.37)		(−0.60)	(−0.59)
**Morbidity burden**									
Charlson, index (excl. diabetes)	0.188 ***					0.0101	0.215 ***		
			(4.10)			(1.49)			(6.30)
**Socioeconomic proportions**									
Proportion of patients, aged >65			0,05			0.00797			−0.0498
			(1.04)			(0.82)			(−0.60)
Proportion of unemployed patients			–0.00159			−0.0121			−0.114
			(−0.02)			(−0.73)			(−1.09)
Patient family income, (DKK)	1.29e-07 **			−1.84e-08				1.61e−08	
			(−3.10)			(−1.65)			(0.19)
Proportion of single patients			0.0727			–0.00601			−0.08
			(1.47)			(−0.48)			(−1.10)
Year & GP fixed effects	Y	Y	Y	Y	Y	Y	Y	Y	Y
Mean outcomes (2008)	0.100	0.100	0.100	0.011	0.011	0.011	0.348	0.348	0.348
R^2^	0.293	0.294	0.338	0.077	0.077	0.080	0.643	0.643	0.657
N	3871	3871	3871	3871	3871	3871	3871	3871	3871

Note: Table shows the effect of point-of-care Testing (POCT) of HbA1c on diabetes-related admissions, ACSC admissions and ambulatory care visits. POCT * I^year^ is the interaction between the treatment variable POCT and I^year,^ which is one from year of introduction onwards and zero before. Year and clinic fixed effects are included in all models. Control variables are all at the level of the clinic. T-statistics in parentheses. GP: General Practice. R^2^: R-squared. Significance levels: ** *p* < 0.01 and *** *p* < 0.001.

**Figure A1:**
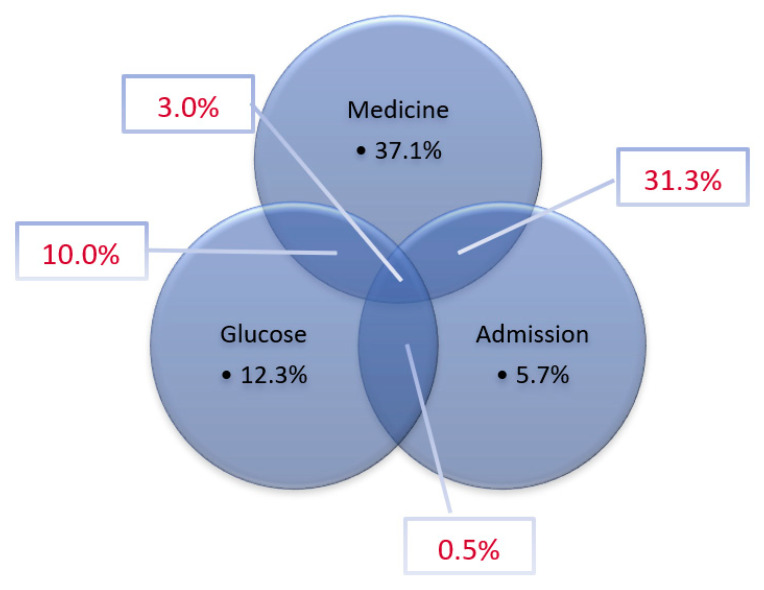
Venn diagram of inclusion criteria for the capital region of Denmark. Note: Figure shows the distribution of the three criteria for which the patients were included in the T2 diabetes population.

## Appendix A

To estimate the effect of POCT in general practice we used a DID framework with a continuous treatment variable and included GP and year fixed effects as well as time-varying control variables. Hence, we estimated the following fixed effect regression model [20,23,24]:

(A1) $$y_{it}=\delta C_{it}I_{it}+\beta x_{it}+\theta_{t}+GP_{i}+\mu_{it}$$

where $y_{it}$ represents our outcome measures of hospital activity for clinic i=1,…,N in year t=2006,…,2012, $C_{it}$ is a continuous treatment variable measuring the share of diabetes patients at $GP_{i}$ treated with POCT in year t, $I_{it}$ is a shift variable which turns to one in the flexible introduction years (2009–2012) in which $GP_{i}$ introduces POCT and hence, $C_{it}I_{it}$ is the interaction of interest representing the treatment effect of POCT. $x_{it}$ is a vector of observed time-varying control variables, $\theta_{t}$ represents year fixed effects that captures time trends common to both treatment and control groups. $GP_{i}$ captures GP clinic level time-invariant unobserved effects (i.e., GP fixed effects).

To study the effects over time relative to the introduction year we used an event study framework. The event study was conducted through regression Equation (A2) which estimates the effects of POCT for the years before and after the introduction of POCT:

(A2) $$\begin{array}{l} y_{it}=\alpha +\overset{-2}{\underset{k=k_{i}^{min}}{\sum }}\beta_{k}^{Before}C_{it}1\left[t-T_{i}=k\right]\\ \;+\overset{k_{i}^{max}}{\underset{k=0}{\sum }}\beta_{k}^{After}C_{it}1\left[t-T_{i}=k\right]+\gamma x_{it}+\theta_{t}+GP_{i}+\mu_{it} \end{array}$$

The variables $y_{it},\;C_{it},\;x_{it},\;\theta_{i},\;GP_{i}$ were defined for Equation (A1), $T_{i}\in \left[2009;2012\right]$ denotes the year where each $GP_{i}$ introduces POCT and $t_{i}-T_{i}=k_{i}$ ∈ [−4;2] represents the number of years before and after $GP_{i}$ introduced POCT(see Table 2). Therefore, the definition of what is before and after becomes dynamic. and the range for $k_{i}^{min}\in$ [−4;−3] and $k_{i}^{max}\in$ [0;2] depend on the year of introduction. The indicator function 1[·] is one if $t-T_{i}$ equals k (shown in Table 2) and zero otherwise. Thus, $\beta_{k}^{Before}$ in the first summation estimates the difference in outcomes between control and treatment for each year (k) *Before* the introduction of POCT and $\beta_{k}^{After}$ in the second summation estimates the effect of POCT on outcomes for each year (k) *After* the introduction of POCT.

As an example, a GP clinic that introduced POCT in 2010 is considered. Hence, $T_{i}=2010$, $C_{it}$ is zero before 2010 and equal to the share of the clinic’s patients with diabetes receiving at least one POCT in the subsequent years. The GP clinic will be part of the control group in the Years 2006 $\left(k_{i}=-4\right),\;$2007 $\left(k_{i}=-3\right)$ and in 2008 $\left(k_{i}=-2\right).\;$2009 is the reference year for $GP_{i}$ and in 2010 $\left(k_{i}=0\right)$, 2011 $\left(k_{i}=1\right)$ and 2012 $\left(k_{i}=2\right)$ $GP_{i}$ will be part of the treatment group. Hence, in this case, $GP_{i}$ has $k_{i}^{min}=-4$ and $k_{i}^{max}=2$ and will be included in three pre-introduction estimates ($\beta_{-4}^{Before}$, $\beta_{-3}^{Before}\;\mathrm{and}\;\beta_{-2}^{Before}$) and three post-introduction estimates ($\beta_{0}^{After}$, $\beta_{1}^{After}\;\mathrm{and}\;\beta_{2}^{After}$).

The flexible reference year is always the year before $GP_{i}$ introduces POCT, k=−1. This year (rather than k=0) was used to ensure that the GP did not use the equipment in the reference year. All estimates should be interpreted relative to that flexible reference year. As $GP_{i}$ introduces POCT at some point throughout the year k=0, $\beta_{0}^{A}$ should be interpreted in that context; effects may not have occurred fully yet.

## Appendix C

The following ICD10 codes from the hospital sector were used to define the sample of T2 diabetes patients: E11.0 coma, E11.1 ketoacidosis, E11.2 kidney complications, E11.3 eye complications (potential outcome - blind), E11.4 neurological complications (difficult to feel or pain), E11.5 complications in peripheral veins, E11.6 other complications, E11.7 multiple complications, E11.8 complications without specification and E11.9 without complications. E12 diabetes related to malnutrition, E13 related to other diabetes and E14 without specification were also subdivided across the above-mentioned subgroups 1–9. E12 is not used very often because there are relatively few of these patients. E13 and E14 are unspecific and often not used. O24 is related to pregnancy, birth and maternity. O24.0 is a T1 pregnancy, O24.1 is T2 pregnancy, 024.2 is pregnancy related to malnutrition and D024 is unspecified. Patients with no other diabetes-related issues than DO244 (gestational diabetes pregnancy, birth and maternity) were excluded. H360* is a diabetes-related change in eyes. It is also often used by specialists in primary care. The 11.3 code is used by hospital staff.
